# Epitaxial SiC Dosimeters and Flux Monitoring Detectors for Proton Therapy Beams

**DOI:** 10.3390/ma16103643

**Published:** 2023-05-10

**Authors:** Mara Bruzzi, Enrico Verroi

**Affiliations:** 1 Dipartimento di Fisica e Astronomia, Università degli Studi di Firenze, Via G. Sansone 1, 50019 Sesto Fiorentino, FI, Italy; 2 I.N.F.N. Sezione di Firenze, Via G. Sansone 1, 50019 Sesto Fiorentino, FI, Italy; 3 Consorzio Interuniversitario Nazionale per la Scienza e Tecnologia dei Materiali (INSTM), Via G. Giusti 9, 50121 Firenze, FI, Italy; 4 Trento Institute for Fundamental Physics and Applications, National Institute of Nuclear Physics (TIFPA), Via Sommarive, 14, 38123 Povo, TN, Italy

**Keywords:** SiC, epitaxial films, radiation detectors, dosimeters, proton therapy

## Abstract

The exceptional optoelectronic properties and high radiation resistance of epitaxial silicon carbide make this material attractive for high-energy beam dosimetry and radiation monitoring, especially when strict requirements such as high signal-to-noise ratios, high time and spatial resolutions and low detectivity levels are required. A 4H-SiC Schottky diode has been characterized as a proton-flux-monitoring detector and dosimeter under proton beams for proton therapy. The diode was composed of an epitaxial film grown on 4H-SiC n^+^-type substrate equipped with a gold Schottky contact. The diode was embedded in a tissue-equivalent epoxy resin and then characterized in terms of capacitance vs. voltage (C-V) and current vs. voltage (I-V) characteristics in the dark in the range of 0–40 V. The dark currents at room temperature are in the order of 1 pA, while the doping and active thicknesses extracted from the C-V are 2.5 × 10^15^ cm^−3^ and 2–4 μm, respectively. Proton beam tests have been carried out at the Proton Therapy Center of the Trento Institute for Fundamental Physics and Applications (TIFPA-INFN). They have been carried out with energies and extraction currents of 83–220 MeV and 1–10 nA, respectively, as typical for proton therapy applications, corresponding to dose rates in the range of 5 mGy/s to 2.7 Gy/s. The I-V characteristics measured under proton beam irradiation at the lowest dose rate showed a typical diode photocurrent response and a signal-to-noise ratio well above 10. Investigations with null bias evidenced a very good performance in terms of the diode’s sensitivity, fast rise and decay times and response stability. The diode’s sensitivity was in agreement with the expected theoretical values, and its response was linear throughout the whole investigated dose rate range.

## 1. Introduction

Radiation detectors that are routinely applied in radiotherapy are mainly based on ionization chambers and semiconductor devices. Despite their widespread and diffuse application, the performance of these well-established radiation detectors is still affected by limitations. Intense research activity is underway for the development of innovative semiconductor materials and devices, aiming to match the numerous, stringent requirements associated with the various particle beams and radiation fields, as well as their application environments [1,2,3,4,5,6,7]. 

The working principle of a semiconductor detector is based on the generation of electron–hole (e–h) pairs in the material bulk by the incoming radiation. The generated, charged carriers are accelerated by an electric field and collected by electrodes, forming the electric signal of the device. Usually, the electric field is created by applying a constant voltage drop across two electrodes in either a transverse or planar geometry. In the case of diodes, it may be generated by the presence of a Schottky barrier or a p-n junction. The electric signal of the detector, associated with the incoming radiation, may be collected in either the pulse or current mode, depending on the particular application. The output of the pulse-mode detector is a train of voltage pulses, each corresponding to a detected particle; it may be thus regarded as a single-event process. The voltage signal is then amplified and collected as a histogram through a shaping amplifier and a multichannel analyzer. Current-mode operation, which is generally adopted in real-time dosimetry, consists of monitoring the current signal continuously during irradiation as a function of time, followed by integration at sampling intervals to obtain the collected charge. For proper monitoring, the collected charge should be linear with the dose and, therefore, the current signal should be linearly dependent on the dose rate. The sensitivity of the device, *S*, is generally defined as the slope of the current vs. the dose rate linear function. The dosimetric sensitivity per unit active volume is defined as [6]:

(1) $$S_{V}=\frac{q\rho }{E_{i}}$$

where q is the electronic charge, ρ is the mass density, and E_i_ is the ionization energy required to create an e–h pair. In general, low-bandgap semiconductor materials are characterized by lower ionization energies [8]. At the same dose, they provide high concentrations of generated e–h pairs and consequently higher signals. On the other hand, the low bandgap is responsible for a high thermal generation of e–h pairs in the dark, resulting in high leakage currents and noise. This is the case, e.g., of silicon, which may be affected by an increase in the leakage current at the accumulated dose due to presence of radiation-induced traps [6]. Conversely, high-bandgap materials, characterized by low leakage currents during exposure to radiation, may guarantee lower detection limits and intrinsic radiation hardness [9]. The best representatives of low-bandgap materials for high-energy radiation detection are germanium and silicon [6], while the most widely studied high-band gap materials for the same application are diamond and SiC [7,8,9,10,11,12,13,14,15]. Single-crystal diamond devices of a high morphological quality are already commercially available as radiotherapy dosimeters [15]. Nonetheless, they are characterized by small linear dimensions (of the order of a few millimeters), a geometrical limitation due to the size of the initial seeds used in the epitaxial Chemical Vapor Deposition (CVD) growth technique. Conversely, SiC epitaxial wafers are now routinely grown with large diameters and a high morphological quality [16]. With respect to diamond, SiC is also characterized by a higher sensitivity per unit volume, $S_{V}=$ 411 nC/(Gy mm^3^) compared to 217 nC/(Gy mm^3^) [6,17]. Within the class of high-bandgap materials, it is worth mentioning lead halide perovskites, characterized by a bandgap similar to that of SiC with an even higher sensitivity per unit volume. CsPbCl_3_ and CsPbBr_3_ single crystals and films have recently been tested as beam-monitoring detectors and dosimeters for high-energy particle beams, with promising results [18,19,20]. Nonetheless, devices based on these materials are still in a preliminary stage of research and development [21,22], while epitaxial 4H-SiC Schottky diodes rely on a mature and well-established technology. In the past, the dosimetric properties of 4H-SiC diodes were tested under a Co^60^ source [10], under a 6 MV photon beam from a linear accelerator, and under a 22 MeV electron beam and UV photon fluxes [23]. Very little literature has focused on 4H-SiC Schottky diodes as radiation detectors for hadron therapy applications. Recently, SiC detectors were tested under a 62 MeV proton beam [24], a carbon beam [25] and high-intensity ion beams [26]. This paper aims to explore the application of an epitaxial 4H-SiC Schottky diode as a dosimeter and real-time monitoring detector under proton beams within ranges typical for proton therapy. In particular, this paper focuses on the operative condition of null bias, which is of interest for in vivo applications and has never been investigated before with respect to proton beams. 

## 2. Materials and Methods

**Figure 1:**
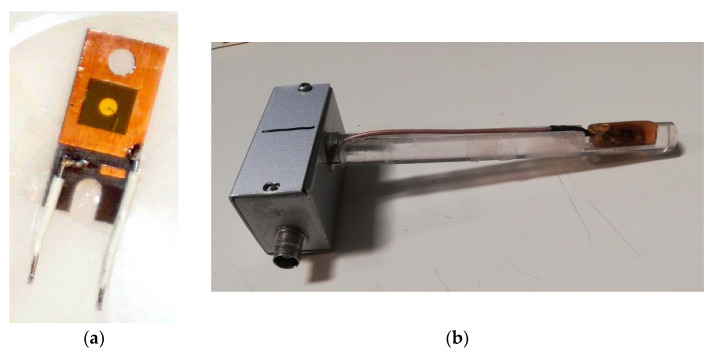
(**a**) The epitaxial 4H-SiC Schottky diode mounted on a printed circuit board and wire- bonded at the front contact. (**b**) Same device embedded in epoxy resin and mounted on the PMMA finger.

Our device is a Schottky diode fabricated by Alenia Systems, Roma, Italy, on 4H-SiC epitaxial wafers. The material consists of an n-type, 4H-SiC epitaxial layer that is 30 μm thick, grown on an n^+^-type substrate of 4H-SiC (thickness: 360 μm), with a nitrogen concentration of 6.8 × 10^18^ cm^−3^. An n^+^-type buffer layer, being 1 μm thick, lies between the epitaxial layer and the substrate. The Schottky contact, being circular with a 2 mm diameter, is a 1000 Å thick gold layer deposited on the epitaxial layer. A Ti/Pt/Au ohmic contact is evaporated on the back surface. The device contact has no guard ring structure. A picture of the 4H-SiC Schottky diode is shown in Figure 1a. The SiC diode is mounted on a miniature printed circuit board (PCB) and wire-bonded on the front contact. It was embedded in epoxy resin to prevent a possible contribution of the ionized air close to the electrodes to the electrical signal. The epoxy resin was characterized using a computerized tomography (CT) scanner previously calibrated to determine its electronic density relative to water, ρ, and its average effective atomic number, Z_eff_. The epoxy was found to be almost tissue-equivalent, with ρ = 1.13 and Z_eff_ = 6.24. The device embedded in resin was then mounted on a PolyMethilMetacrylate (PMMA) finger, as shown in Figure 1b.

Before proton irradiation, we performed electrical tests of the 4H-SiC Schottky diode embedded in the epoxy resin and mounted on the PMMA finger at room temperature. The capacitance vs. voltage (C-V) characteristics and current vs. voltage (I-V) characteristics were investigated at T = 20 °C by means of an HP 4182 A LCR meter at 1 kHz and a Keithley 6517 A high-impedance source/meter, respectively. The latter instrument allows for a low current input amplification, with an ultimate resolution of 10 aA in the pA range and preamplifier settling times of 15 ms in the nA range. 

**Figure 2:**
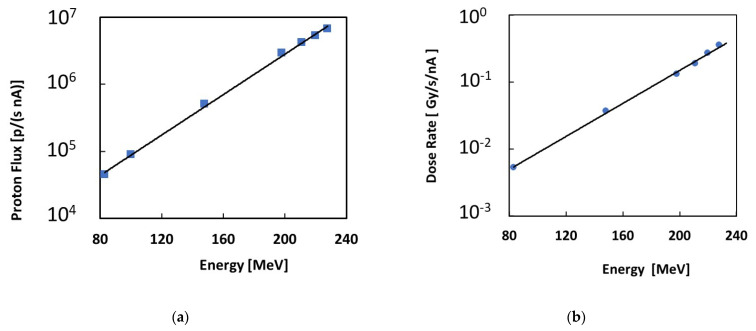
(**a**) Proton fluxes impinging on the detector area per unit extraction current, plotted as a function of the proton beam energy; (**b**) dose rates per unit extraction current plotted as a function of the proton beam energy. Exponential guidelines are added to the plots.

Then, the device was tested at the Trento Proton Therapy Center of Trentino Healthcare Agency (Azienda Provinciale per i Servizi Sanitari—APSS, Italy) by means of a cyclotron (IBA, Proteus 235) serving the experimental area [27]. The used proton beam energies were in the range of 83–220 MeV and the studied extraction currents were in the range of 1–10 nA. The proton beam spot profiles followed a gaussian function [27]:

(2) $$f(r,\mu ,\sigma )=\frac{1}{\sqrt{2\pi }\sigma \left(E\right)}\int_{0}^{r}\exp\left[-\frac{{\left(r-\mu \right)}^{2}}{2{\sigma (E)}^{2}}\right]2\pi rdr$$

where μ and *σ* refer, respectively, to the isocenter position and the standard deviation of the Gaussian, while *r* is the radial distance from the isocenter where the detector was placed. In particular, [27] reports that the standard deviation *σ* is a function of the proton energy for a 1 nA extraction current. We calculated the proton flux per unit (1 nA) extraction current impinging on the detector area as the fraction of protons that actually reached the detector. This was obtained by integrating the beam profile given in Equation (2) with the area of our detector, with a radius *r* = 1 mm and μ=0 isocentre position. The resulting proton flux data are plotted as a function of the proton beam energy in Figure 2a. A best-fit exponential trend is also shown as a guideline. With the precision alignment laser system, the maximum positioning error of the sample can lead to an error of 5% in the case of the narrowest beam. 

The dose rates used during the proton beam tests were measured using a Markus chamber, PPC05, from IBA Dosimetry, Germany. Figure 2b shows the measured dose rates per unit extraction current plotted as a function of the proton beam energy. 

Tests were carried out under continuous irradiation with applied reverse voltages in the range of 0–40 V and the device readout in the current mode. Then, the current signal was measured over a train of long proton beam pulses with different extraction currents and the same proton beam energy. The null bias operative condition was considered. The same test was repeated with different proton energies in the range of 83–220 MeV. During the measurements, the electrometer was set in the auto-range to measure the current, always with the highest sensitivity allowed by the instrument in the different current ranges. The time sampling interval used during the measurements was approximately 0.2 s. 

## 3. Experimental Results

**Figure 3:**
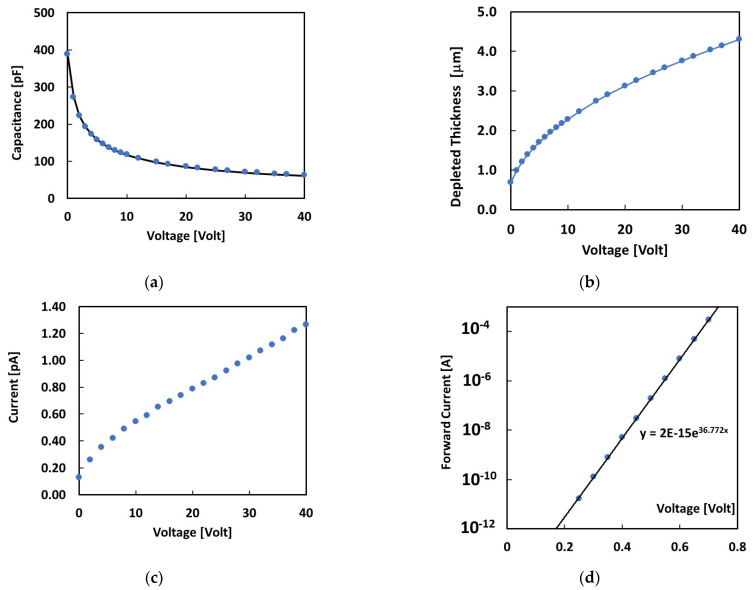
Electrical characteristics of the 4H-SiC Schottky diode embedded in the epoxy resin measured in the dark: (**a**) capacitance vs. voltage and best fit using Equation (1); (**b**) thickness of the depleted region as a function of the voltage; (**c**) reverse current vs. voltage; (**d**) forward current voltage characteristics and exponential best fit.

Before the proton beam tests, we performed electrical tests of the 4H-SiC Schottky diode embedded in the epoxy resin and mounted on the PMMA finger. The capacitance–voltage (C-V) characteristics measured at 20 °C are shown in Figure 3a. The best fit of the C-V shown in the plot was obtained by considering the typical C-V relationship for diodes [28]:

(3) $$C\left(V\right)=\sqrt{\frac{2\varepsilon_{0}\varepsilon_{r}(V_{rev}+V_{bi})}{qN}}$$

With a *V_bi_* = 1.2 V built-in potential, *ε_r_* = 9.7 relative dielectric permittivity of SiC, and *N* = 2.46 × 10^15^ cm^−3^ effective doping concentration of the epitaxial layer, the free parameter of this best-fit procedure. Then, the thickness of the depletion region, W, was determined for each applied voltage from the measured capacitance using the following expression: $C=\frac{\varepsilon_{0}\varepsilon_{r}}{W}A$, with *A* as the detector active area. Figure 3b reports the thickness obtained as a function of the voltage. Its value is in the range 0.7–4.2 μm for the reverse voltages investigated in this work (0–40 V). The reverse current–voltage (I-V) characteristics measured in the dark are shown in Figure 3c. A very low reverse current, less than 2 pAs, was observed within the whole investigated voltage range.

Figure 3c shows the current measured as a function of the forward voltage. The forward current was fitted to an exponential function, this having been well-verified over eight decades. The exponential best fit was obtained with *n* ≈ 1 as the ideality factor, indicating a negligible contribution of other electronic transport mechanisms across the barrier to the thermionic current [28]. From the value of the current intercept on the voltage axis of the forward I-V characteristics, we evaluated the saturated ideal reverse current and the zero-voltage barrier height [28]:

(4) $$I_{s}=AA^{*}T^{2}e^{-\frac{q\varphi_{b0}}{KT}}$$

with A* = 137 A/cm^2^ K^2^, the effective Richardson constant, and *φ_b_*_0_ zero-voltage barrier height [25]. The values thus extracted are *I_s_* = 2 × 10^−15^ A and *φ_b_*_0_ = 1.2 V.

**Figure 4:**
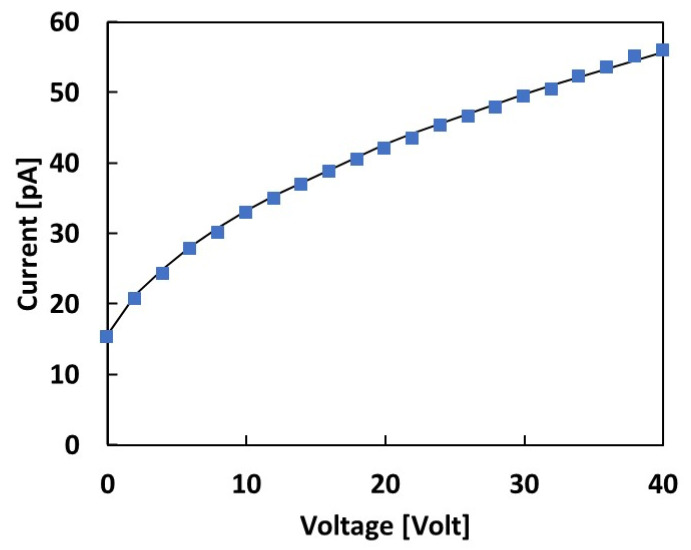
Current–voltage characteristics of the SiC device under exposure to an 83 MeV proton beam with a 1 nA extraction current. The best fit shows a square root dependence on the voltage.

Figure 4 shows the I-V characteristics measured with the SiC device in the range of 0–40 V under continuous irradiation with an 83 MeV proton beam and 1 nA extraction current. We note that these are the conditions of the lowest dose rate and proton flux investigated in this study. The data were best-fitted considering a diode-like dependence of the current with the square root of the bias, indicating a typical diode-like dependence of the electrical conductivity. 

Comparing the I-V plot measured during irradiation under the 83 MeV proton beam at a 1 nA extraction current with the dark I-V characteristics shown in Figure 3c, we observe that even in these irradiation conditions, corresponding to the lowest proton flux and dose rate, the current, at any bias, was more than ten times higher than the dark current. These measurements evidence a signal-to-noise ratio, S/N, that is always higher than 10. In particular, the condition of null bias is of interest for in vivo applications, and it represents the lowest S/N limit. Thus, as already mentioned, in our further measurements, we focused on the performance of our device in null bias conditions. 

**Figure 5:**
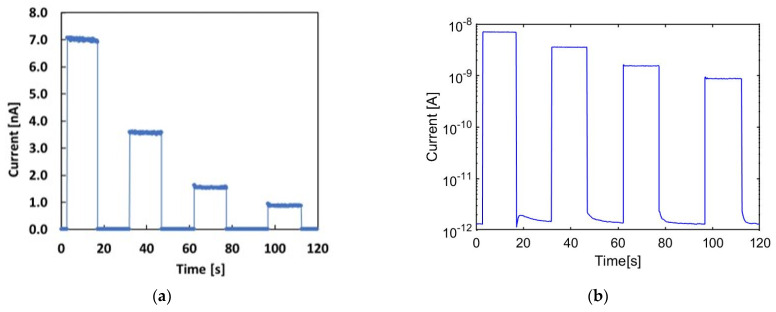
(**a**) Current signal (linear plot) vs. time measured with the SiC device during four pulses of the 220 MeV proton beam at different extraction currents, respectively: 10, 5, 2 and 1 nA. (**b**) The same data shown in a semilogarithmic plot.

The plots in Figure 5a,b (same data shown on linear and log scales) show the response of the 4H-SiC Schottky diode at null bias to a 220 MeV proton beam. 

Specifically, 20 s long pulses were delivered each time at different extraction currents: 10, 5, 2 and 1 nA. The linear plot shows the signal stability when the proton beam is on: a relative variation of less than 1% is observed during each exposure. The logarithmic plot shown in Figure 5b shows the fact that the low dark current rapidly settles each time the beam is switched off. When the beam is on, the signal is in the range of nA, being three orders of magnitude higher than the signals in the dark. 

**Figure 6:**
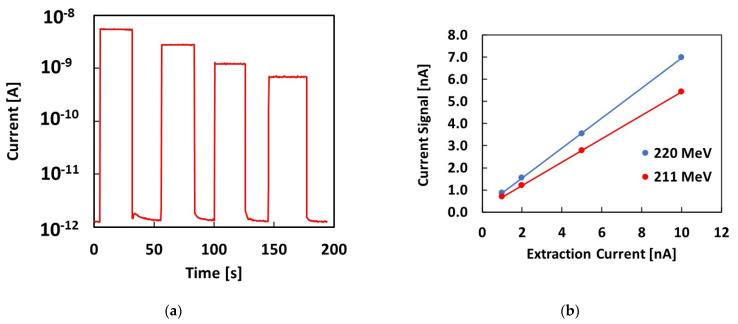
(**a**) Current signal vs. time measured with the 4H-SiC Schottky diode when four pulses of a 211 MeV proton beam and different extraction currents of 10, 5, 2 and 1 nA are delivered; (**b**) the plateau average current signal measured upon the delivery of 220 MeV and 211 MeV proton beams, respectively, plotted as a function of the extraction current and linear best fits.

The same experimental investigation was carried out with other proton energies. As an example, Figure 6a shows the current signal measured during irradiation with a 211 MeV proton beam and 10, 5, 2 and 1 nA extraction currents. The logarithmic plot shows that the dark current, when beam is switched off, is always in the pA range, as shown in Figure 5b for the 220 MeV proton beam. Figure 6b shows the current signal measured when the beam is on as a function of the extraction current for both proton energies. The linear dependence of the current signal on the extraction current is verified for both proton energies.

## 4. Discussion

The reverse I-V characteristics shown in Figure 4 allow us to evaluate the total active thickness of the dosimeter under proton irradiation. To this end, we consider the photoconductive response due to a partially depleted semiconductor diode under constant radiation flux exposure. Assuming a uniform generation rate of hole–electron pairs, *G*, throughout the semiconductor material, as is the case for the proton beams considered in this study, the photoconductive response is mainly due to two different contributions [6]. A fast response is due to those carriers generated in the depleted region of the device, W, that will be accelerated by the electric field and collected by the electrodes. A slower contribution is also made by carriers generated in the neutral bulk, which may reach the depleted region via diffusion. If the collection time is long enough to include both contributions, as in our case, the photocurrent will be given by the following expression [6]:

(5) $$I=qA\left(W+L\right)G$$

where *q* is the electronic charge, *W* is the depleted thickness of the junction, *L* is the minority diffusion length, and *G* is the generation rate of the e–h carriers during radiation exposure. To best-fit our data, *L* and *G* are considered as free parameters, while *W* denotes values obtained from the C-V characteristics, measured in the dark at the same bias (see Figure 3b). The best fit shown in Figure 4 is then obtained, with *G* = 2.2 × 10^13^ cm^−3^/s and *L* = 0.8 μm. This latter corresponds to a minority carrier lifetime *τ* of approximately 2 ns, taking into account the following relationship [28]:

(6) $$L=\sqrt{D\tau }$$

with *D*, the diffusivity for holes, being *D* = 2.98 cm^2^/s, obtained assuming μ*_h_* = 115 cm^2^/V·s.

**Figure 7:**
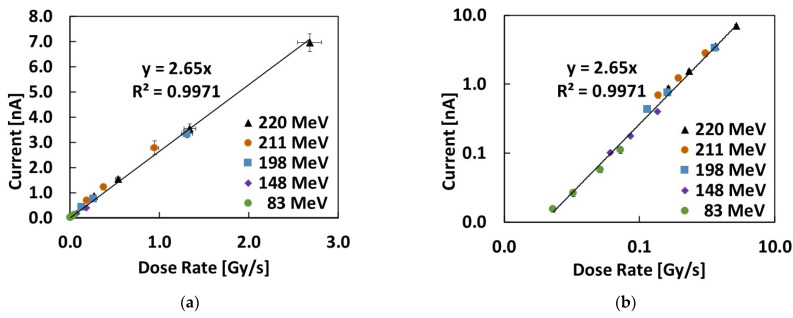
Current signals measured during irradiation with the proton beams using the 4H-SiC Schottky diode at null bias. Data are plotted as a function of the dose rate: (**a**) linear and (**b**) log–log plot evidencing a linear dependence. The linear best fit is added to the plots.

**Figure 8:**
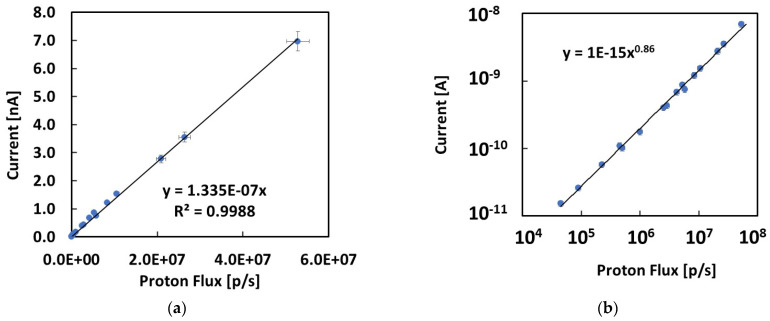
Current signals measured with the SiC device at null bias plotted as a function of the proton fluxes impinging on the detector: (**a**) linear plot and linear best fit; (**b**) log–log plot showing the best fit as a power law.

The current signals measured by the 4H-SiC Schottky diode at null bias for all the investigated proton energies and extraction currents are plotted in Figure 7 and Figure 8, respectively, as a function of the dose rate and of the proton flux impinging on the detector area. These latter were obtained using the data plotted in Figure 2a,b by multiplying the values by the used extraction currents.

Groups of data collected at the same proton energies and different extraction currents are shown with the same symbols in Figure 7a,b. The linear best fit of the whole dataset is also shown in the two plots. In particular, Figure 7b demonstrates how well the linear dependence holds throughout the entire investigated dose rate range, covering almost 3 orders of magnitude (the tested dose rates are from 5.3 mGy/s to 2.68 Gy/s). 

The slope of the linear best fit may be considered as the sensitivity of the 4H-SiC diode at null bias: *S* = 2.65 nC/Gy. This sensitivity may be related to the known theoretical value per unit volume *Sv* = 411 nC/(Gy mm^3^) for SiC [17], according to:

*S* = *S_v_AW*, (7)

where the product *AW* is the active volume of the diode at null bias. Taking *A* = 3.14 mm^2^ as the Schottky contact electrode area, we obtain a total active thickness at null bias ≈ 2 μm. This value is highly comparable with the evaluation of *L* + *W* = 1.6 μm obtained by fitting the C-V and I-V characteristics shown in Figure 3 and Figure 4, as discussed earlier. 

Finally, Figure 8a shows the current signal measured at null bias as a function of the proton fluxes impinging on the detector area during irradiation. Here, we took all the combinations of proton energies and extraction currents measured in this study. The fluxes impinging on the detector active area were from 4.50 × 10^4^ p/s to 5.28 × 10^7^ p/s, covering almost 3 orders of magnitude. Figure 8a shows that the linear response with the proton flux holds especially in the highest range investigated in this work. Figure 8b shows same data in a log–log plot, illustrating possible deviations occurring throughout all three investigated orders of magnitude. The data show an almost linear trend over the three orders of magnitude. Nonetheless, a deviation from linearity occurs, particularly within the lowest proton flux range, below 10^5^ p/s. For this reason, the total set of data may be better described by a power law dependence with a power factor a = 0.86. This experimental evidence may be due to errors in the positioning of the SiC device, which has a very small area with respect to the standard deviation of the Gaussian proton beam distribution. The phenomenon will be investigated in detail in forthcoming works.

## 5. Conclusions

The 4H-SiC is a semiconductor material with a mature manufacturing technology and attractive electrical transport properties for the detection of high-energy particle beams. In this work, a 4H-SiC Schottky diode embedded in a tissue-equivalent resin was tested as a real-time monitoring detector and dosimeter under proton beams with energies (83–220 MeV) and extraction currents (1–10 nA) typical of proton therapy applications. Our experimental results evidenced the excellent performance of the SiC epitaxial detectors in terms of their response stability, signal-to-noise ratios and linearity, with dose rates of over three order of magnitude. The dark current of the device, which is always below 2 pA, allows one to achieve S/N ratios > 10 at dose rates of 3 orders of magnitude, even when keeping the device at null bias. The response of the diode to variable dose rates in the ranges of 5 mGy/s–2.7 Gy/s was found to be linear. The sensitivity measured at null bias is in agreement with the expected theoretical values. The dependence of the signal on the proton flux range was also tested based on three orders of magnitude, from 4.5 × 10^4^–5.3 × 10^7^ p/s. A slight non-linearity was observed in the lowest range of fluxes, whose origin will be investigated in forthcoming works. 

## Data Availability

Data supporting reported results can be found within the paper itself.
